# Assessment of hypoxia and oxidative-related changes in a lung-derived brain metastasis model by [^64^Cu][Cu(ATSM)] PET and proteomic studies

**DOI:** 10.1186/s13550-023-01052-8

**Published:** 2023-11-25

**Authors:** Jade Fantin, Jérôme Toutain, Elodie A. Pérès, Benoit Bernay, Sarina Maya Mehani, Charly Helaine, Mickael Bourgeois, Carole Brunaud, Laurent Chazalviel, Julien Pontin, Aurélien Corroyer-Dulmont, Samuel Valable, Michel Cherel, Myriam Bernaudin

**Affiliations:** 1 Université de Caen Normandie, CNRS, Normandie Univ., ISTCT UMR6030, GIP CYCERON, F-14000 Caen, France; 2 Université de Caen Normandie, Normandie Univ., US EMerode, Plateforme Proteogen, F-14000 Caen, France; 3 CRCI2NA, INSERM UMR1307, CNRS-ERL6075, Université d’Angers, Université de Nantes, F-44000 Nantes, France; 4 GIP ARRONAX, F-44800 Saint-Herblain, France; 5 Medical Physics Department, CLCC François Baclesse, F-14000 Caen, France

**Keywords:** Brain metastasis, Lung cancer, Hypoxia, HIF, Oxidative stress, Cu-ATSM, Imaging, Proteomic, Preclinical, Rodent, Copper-64, PET

## Abstract

**Background:**

Brain metastases (BM) are the most frequent malignant brain tumors. The aim of this study was to characterize the tumor microenvironment (TME) of BM and particularly hypoxia and redox state, known to play a role in tumor growth and treatment resistance with multimodal PET and MRI imaging, immunohistochemical and proteomic approaches in a human lung cancer (H2030-BrM3)-derived BM model in rats.

**Results:**

First, in vitro studies confirmed that H2030-BrM3 cells respond to hypoxia with increasing expression of HIF-1, HIF-2 and their target genes. Proteomic analyses revealed, among expression changes, proteins associated with metabolism, oxidative stress, metal response and hypoxia signaling in particular in cortical BM. [^64^Cu][Cu(ATSM)] PET revealed a significant uptake by cortical BM (*p* < 0.01), while no uptake is observed in striatal BM 23 days after tumor implantation. Pimonidazole, HIF-1α, HIF-2α, CA-IX as well as GFAP, CTR1 and DMT1 immunostainings are positive in both BM.

**Conclusion:**

Overall, [^64^Cu][Cu(ATSM)] imaging and proteomic results showed the presence of hypoxia and protein expression changes linked to hypoxia and oxidative stress in BM, which are more pronounced in cortical BM compared to striatal BM. Moreover, it emphasized the interest of [^64^Cu][Cu(ATSM)] PET to characterize TME of BM and depict inter-metastasis heterogeneity that could be useful to guide treatments.

**Supplementary Information:**

The online version contains supplementary material available at 10.1186/s13550-023-01052-8.

## Background

Brain metastases (BM) are the most frequent malignant brain tumors. They occur 3–10 times more than primary brain tumors [1]. Twenty to thirty percent of patients with solid cancers will develop BM with approximately 50% of cases coming from lung cancer, 15% from breast cancer, 10% from renal cell carcinoma and 9% from melanoma [2]. The median survival can vary from 3 to 47 months and depends on type of primary tumor and prognostic factors [3]. Treatments for BM include symptomatic treatments and when possible, surgical resection followed by radiotherapy (RT), including stereotactic radiosurgery (SRS) and/or whole-brain RT (WBRT) [4, 5]. Presently, treatments for BM do not consider the tumor microenvironment (TME), which plays a crucial role in the treatment response. Among TME features of solid cancers, hypoxia is known to be a poor prognostic factor, associated with tumor progression and resistance to cancer treatments including RT in many cancers [6, 7]. One of the main cellular responses to hypoxia is the stabilization of the transcriptional factors hypoxia-inducible factors (HIFs) also known to be a poor prognostic factor and associated with tumor aggressiveness [8]. However, only few studies focused on TME of BM and in particular on hypoxia. First, Berghoff et al. [9] showed, in patients with BM from lung cancer, that low HIF-1α expression is associated with an increased lifespan than patients with high HIF-1α expression. Two additional clinical studies showed that HIF-1α expression is higher in BM compared to their matching primary tumors for lung, breast and colorectal cancers and associated with tumor proliferation and decrease in overall survival [10, 11]. Moreover, we showed elevated expression of HIF-1α and carbonic anhydrase IX (CA-IX), a well-known HIF-target gene in biopsies of BM from lung cancer [12, 13]. In the same study, we evidenced by [^18^F]FMISO positron emission tomography (PET) and oxygen saturation (Sat-O_2_) MRI imaging, the presence of hypoxia in the TME of BM as well as inter-metastasis heterogeneity in lung cancer-derived preclinical models of BM [13]. These data suggest the relevance of detecting hypoxia in BM to refine treatment strategy and improve patient prognosis.

Many radiotracers have been proposed for the detection of hypoxia by PET and in particular [^18^F]FMISO as reviewed in [14, 15]. However, this radiotracer is mostly sensitive to severe hypoxia (< 10 mmHg) which does not allow the detection of more moderate hypoxia. In recent years, [^64^Cu][Cu(ATSM)] has been suggested as a promising radiotracer in the detection of hypoxia as well as oxidative stress [15–17]. Indeed, Cu(II) has a low redox potential allowing its stability in normal tissues, and ATSM confers lipophilic properties to facilitate its passage through membranes. Thus, it is rapidly washed out under normal conditions, it will be retained in cells with an over-reducing state, like under hypoxia [18–20]. The mechanism of intracellular retention of radioactive copper is based on the reduction of Cu(II) to Cu(I) which is less soluble and unstable form. Studies have shown that the reduced state of the cells due to mitochondrial dysfunction could also be the cause of Cu(I) production [21]. Therefore, the retention of Cu-ATSM can depend on the redox state of the cells independently of hypoxia [22]. Indeed, even if a positive correlation between [^64^Cu][Cu(ATSM)] and [^18^F]FMISO or HIF-1α has been shown in glioblastoma models [23, 24], other studies highlighted the failure of [^64^Cu][Cu(ATSM)] to correlate with hypoxic markers contrary to [^18^F]FMISO [25]. Moreover, we showed, in a preclinical glioblastoma model, an uptake of [^64^Cu][Cu(ATSM)] in regions with severe hypoxia but also at the periphery of the tumors where staining for pimonidazole, CA-IX and HIF-1α is negative. Interestingly, this latest region showed an increase expression of copper transporters (DMT1 and CTR1) associated to astrogliosis [26].

This study aims to provide, for the first time, [^64^Cu][Cu(ATSM)] PET imaging in BM along with additional knowledge of TME of BM more specifically on hypoxia and redox state using immunohistochemistry and proteomic approaches in the H2030-BrM3 lung-derived BM model. Expression studies of HIFs and their target genes were also performed in vitro in H2030-BrM3 cells to evaluate the ability of these cells to respond to hypoxia. The final goal of this study is to evaluate the interest of [^64^Cu][Cu(ATSM)] PET as global hypoxic/oxidative stress radiotracer that can depict inter-metastasis and/or intra-metastasis heterogeneity that could be of clinical utility to refine treatment strategy.

## Materials and methods

### Cell culture

The human H2030-Br3M adenocarcinomas cells (*KRAS*^G12C^ mutated from MSKCC, Dr. Joan Massagué) that preferentially metastasize to the brain were used. Cells were grown in DMEM (Sigma-Aldrich, France), 1 g/L of glucose supplemented with 2-mM glutamine (Gibco, France), 100-U/mL penicillin, 100-µg/mL streptomycin and 10% fetal calf serum (Eurobio, France) at 37 °C in wet atmosphere. For hypoxic condition, cells were placed at 1% of O_2_ during 3 h–40 h (Ruskinn chamber InvivO_2_ 500, ABE, France).

### Immunocytochemistry

H2030-Br3M cells were plated in 24-well plates on coverslips. One day later, cells were placed in normoxia or hypoxia at 1% of O_2_ during 3 h or 24 h. Cells were fixed with 4% paraformaldehyde (PFA). Non-specific staining was blocked with a solution of 3% bovine serum albumin (BSA) (Sigma-Aldrich, France)—PBS-0.1% Tween (Sigma-Aldrich, France) for 1 h at room temperature. Then, cells were incubated overnight at 4 °C with a primary antibody. The following primary antibodies were used: HIF-1α (1/500; Cell signaling #36,169) and HIF-2α (1/200; Genetex, #30,114) in 1% BSA-PBS-0.1% Tween. The revelation was achieved by an Alexa-555-conjugated anti-rabbit secondary antibody (1/200; Invitrogen, A21428). Cells were counterstained with Hoechst 33,342 (10 µg/mL; Sigma-Aldrich, France) for nuclear staining.

### RT-qPCR

Cells were cultured under normoxia (21% of O_2_) or hypoxia (1% of O_2_) for 40 h. This time of hypoxia was chosen to be close to the in vivo conditions of the microenvironment and to be under chronic hypoxia in order to stabilize the HIF-2 target genes [27, 28]. Extraction of total RNA (ribonucleic acid) was performed using Nucleospin® RNA plus Kit (Macherey–Nagel, France) according to the manufacturer’s protocol. Reverse transcription was performed to obtain complementary DNAs from the RNAs. For each sample, 1 μg of RNA was heated for 5 min at 65 °C in 12 μL containing 1 μL of dNTP (deoxynucleotide tri-phosphate), at 10 mM, and 1 μL of oligo-DT (Oligodeoxythymidine) (500 μg/mL). The reaction mixture was then supplemented with 4 μL of FS buffer (First-Strand Buffer), 2 μL of DTT (Dithiothreitol) (0.1 M), 1 μL of RNAase inhibitor (40 U/L) and 1 μL of M-MLV (Moloney, Murine Leukemia Virus) (200 U/L) and then incubated for 90 min at 37 °C and 15 min at 70 °C. Forward (F) and reverse (R) primers are detailed in Table 1. Assays were run in triplicate on the QuantStudio™ 3 Real-Time PCR System (Applied Biosystems, France). The amplification profile was as follows: hold stage enzyme activation, 95 °C for 3 min; PCR stage 40 cycles: 3 s at 95 °C and 30 s at 60 °C.

**Table 1:** Details of primers for RT-qPCR analysis

Target	Forward	Reverse
*TUBB3*	GAC-CGC-ATC-ATG-AAC-ACC-TTC-AG	AGT-AGG-TCT-CAT-CCG-TGT-TCT-CC
*VEGF-A*	ACT-GCC-ATC-CAA-TCG-AGA-CC	GAT-GGC-TTG-AAG-ATG-TAC-TCG-ATC-T
*SLC2A1*	ATA-CTC-ATG-ACC-ATC-GCG-CTA-G	AAA-GAA-GGC-CAC-AAA-GCC-AAA-G
*CCDN1*	CCT-CTT-CAAC-CTT-ATT-CAT-GGC-TGA	GT-ATC-GTA-GCA-GTG-GGA-CAG-GT
*SERPINE1*	AAG-ACT-CCC-TTC-CCC-GAC-TC	GGC-GTG-GTG-AAC-TCA-GTA-TAG-TT
*CA-IX*	TAT-CTG-CAC-TCC-TGC-CCT-CTG	CAC-AGG-GTG-TCA-GAG-AGG-GTG
*S16*	CTG-GAG-CCA-GTT-CTG-CTT-CT	TCT-GGT-AAT-AGG-CCA-CCA-GG

The PCR was done using 5 μL of cDNA diluted in 15 μL of a mix of a reaction mixture composed by 10 μL of Takyon (Eurogentec), 0.5 μL of forward primer and 0.5 μL reverse primer and 4 μL of H_2_0 RNAase free. Results were analyzed using a comparative method between the fractional cycle number to reach a fixed threshold and the fractional cycle number of S16 gene and expressed using the 2^−ΔCt^ formula.

### H2030-BrM3 lung-derived brain metastasis model

Nude athymic rats (200–250 g, 8 weeks, female, CURB/ONCOModels, Caen) were maintained in specific pathogen-free housing. Rats were manipulated under general anesthesia (5% isoflurane for induction and 2% for maintenance in 70% N_2_O/30% O_2_). Body temperature was monitored and maintained at 37.5 ± 0.5 °C throughout the experiments. For the BM model, rats were placed in a stereotactic head holder, and a scalp incision was performed along the sagittal suture. To investigate potential inter-metastases heterogeneity, two burr holes of diameter 1 mm were drilled in the skull, 3- and 3.7-mm lateral left and right, respectively, to the Bregma. H2030-Br3M cells (5 × 10^4^ cells in 3-μl PBS containing glutamine 2 mM) were injected over 5 min via a fine needle (30G) connected to a Hamilton syringe. The injection sites were the left caudate putamen at a depth of 6 mm and the right cortex at a depth of 2.5 mm. Animals were then followed by anatomical MRI over 24 days period to follow BM development. MRI acquisitions were performed before each PET imaging at D22, D23 and D24.

### Preclinical magnetic resonance imaging (MRI)

MRI scans were performed on a hybrid PET/7T MRI system (Bruker, CYCERON biomedical imaging platform, Caen), once a week to monitor tumor development and before each PET acquisition. For all MRI experiments, rats were under anesthesia (5% isoflurane for induction and 2% for maintenance, in 70% N_2_O/30% O_2_) and were placed in a prone position. Respiration was monitored by a pressure sensitive balloon around the abdomen. After a localizer imaging, an anatomical exploration of the brain was performed using a T2w sequence (RARE, acceleration factor of 8; TR/TE = 5000/62.5 ms; experiments average = 1; 20 contiguous slices; field of view (FOV): 35*35*15; matrix: 192*192*20; resolution: 0.182*0.182*0.75; acquisition time = 2 min). TR and TE are, respectively, repetition time and echo time. A T1 FISP-3D (fast imaging with steady-state precession 3D) sequence (TR/TE = 5/2.4 m; average = 3; FOV: 35*35*50, matrix: 70*70*100; resolution: 0.5*0.5*0.5 resolution) was used just before PET acquisition to generate an attenuation map.

### Positron emission tomography (PET)

[^18^F]FDG was produced by Curium Pharma (France). [^64^Cu][Cu(ATSM)] was provided by the GIP ARRONAX (Nantes, France). PET acquisitions were performed on a PET/7T MRI system (7 Tesla, Bruker, CYCERON, biomedical imaging, Caen). Radiotracers were injected into the caudal vein with an average dose of 27 MBq (20 MBq–34 MBq for [^18^F]FDG and 23 MBq–31 MBq for [^64^Cu][Cu(ATSM)]). Just prior to PET imaging (1 h for [^18^F]FDG and 4 h or 24 h for [^64^Cu][Cu(ATSM)]), T2w anatomical sequence was acquired to observe BM; then, animals were automatically transferred into the PET rings using the ATS system (Bruker) to match PET images with MRI images. Decay corrected PET images were reconstructed by the iterative maximum a posteriori (MAP) algorithm with correction of PVC, PSF, scatter and diffusion. The matrix size of the reconstructed images was 180*180*198 with a FOV of 90*90*99 mm and the resolution of 0.5 × 0.5 × 0.5 mm.

### Imaging data analyses

- *Image processing and analyses* were performed with in-house macros based on the ImageJ software [29]. PET analyses were performed by PMOD 3.0 (Pmod Technologies LLC).
- *MRI tumor volume* was delineated manually on all adjacent T2w slices. Tumor volume was calculated by multiplication of the sum of contiguous tumor surface areas by the slice thickness.
- *MRI/PET coregistration*: All MRI scans were executed such that the various MRI parameters were anatomically registered to each other.
- *Tumor delineation* was performed manually on all adjacent T2w slices. The region of interest (ROI) corresponding to the tumor.
- *PET image analyses*: ROIs defined on T2w MRI were transferred onto all PET images. To quantify [^18^F]FDG and [^64^Cu][Cu(ATSM)] uptakes, the measured tissue activity concentration (counts kBq/mL) was divided by the injected activity in kBq per gram of body weight (kBq/g) to give a standardized uptake value (SUV, g/mL). The SUV in the ROI divided by the value of healthy tissue in the cerebellum to give the relative SUV (rSUV).

### Statistical analyses

*D*ata were analyzed with GraphPad Prism 9.0 software for statistics. The different tests used are detailed in each figure legend. All data are presented as mean ± SD. One sample t-test *vs* theorical value of 1 was used to evaluate rSUV of cortical BM and striatal BM and HIF-target gene expression in hypoxia. Mann–Whitney was used for comparison rSUV between cortical BM and striatal BM, and two-way ANOVA followed by Tukey’s test was used for comparison of tumor volume between cortical BM and striatal BM at D22, D23 and D24.

### Immunohistology

Brains were collected at D24 for the immunohistological analyses from seven different animals. For hypoxia staining, rats were injected with pimonidazole (Hypoxyprobe®-1, Hypoxyprobe Incorporation, USA) at 80 mg/kg i.p., 120 min before the animals were euthanized under deep anesthesia. Then, the rat brains were withdrawn and immediately snap-frozen for subsequent immunohistochemistry. First, slices were post-fixated in PFA 4% for 15 min, then the non-specific binding sites were blocked by 3% BSA %—Tween 0.1%—Triton 0.5% in PBS solution for 90 min at room temperature. The slices were incubated overnight with primary antibodies at 4°C in 1% BSA—Tween 0.1%—Triton 0.5% in PBS solution (Table 2), and the staining was revealed by fluorochrome-conjugated secondary antibodies. Nuclei were counterstained with Hoechst 33,342 (Sigma-Aldrich, 10 μg/mL). Tissue sections were examined at × 10 magnification with fluorescent microscope (Olympus VS 120).

**Table 2:** Details of primary antibodies used for immunohistochemistry

Target	Dilution	Supplier	Reference
CA-IX	1:350	Novus Biologicals	NB 100-417
HIF-1α	1:500	Cell signaling	#36,169
HIF-2α	1:250	GeneTex	#30,114
DMT1	1:200	Abcam	Ab55735
CTR-1	1:500	Novus Biologicals	NB 100-402
Pimonidazole	1:200	Hypoxyprobe Inc.	HP7-1000Kit
GFAP	1:200	DAKO	Z0334

### Proteomic analysis

The cortical and striatal BM were harvested from five different animals at D24 and were frozen before proteomic analysis. Cortex and striatum from three healthy animals were also used for proteomic analyses.

#### Sample preparation and analyses

Tissues were crushed on ice in lysis buffer consisting of 1 M Tris-HCL (pH 7.5), 3 M NaCl, 1% Triton X-100, 0.1% SDS 20% and sterile water. The lysates were then centrifuged for 5 min at 800 g at 4 °C. The supernatants were recovered and stored at -20 °C before assay. Proteins were assayed by the BCA method (BCA (Bicinchoninic Acid) Protein Assay Kit, Thermo Fisher). The plate was incubated at 37 °C for 30 min. Finally, the reading was taken at 562 nm and is related to the standard bovine serum albumin range.

Five µg of each protein extract were prepared using a modified gel-aided sample preparation protocol [30]. Samples were digested with trypsin/Lys-C overnight at 37 °C. For nano-LC fragmentation, protein or peptide samples were first desalted and concentrated onto a µC18 Omix (Agilent) before analysis.

The chromatography step was performed on a NanoElute (Bruker Daltonics) ultra-high-pressure nanoflow chromatography system. Approximatively 200 ng of each peptide sample were concentrated onto a C18 pepmap 100 (5 mm × 300 µm i.d.) precolumn (Thermo Scientific) and separated at 50 °C onto a reversed phase Reprosil column (25 cm × 75 μm i.d.) packed with 1.6-μm C18-coated porous silica beads (Ionopticks). Mobile phases consisted of 0.1% formic acid, 99.9% water (v/v) (A) and 0.1% formic acid in 99.9% ACN (v/v) (B). The nanoflow rate was set at 250 nl/min, and the gradient profile was as follows: from 2 to 30% B within 70 min, followed by an increase to 37% B within 5 min and further to 85% within 5 min and re-equilibration.

Mass spectrometry (MS) experiments were carried out on a TIMS-TOF pro mass spectrometer (Bruker Daltonics) with a modified nano-electrospray ion source (CaptiveSpray, Bruker Daltonics). A 1400 spray voltage with a capillary temperature of 180°C was typically employed for ionizing. MS spectra were acquired in the positive mode in the mass range from 100 to 1700 m/z and 0.60 to 1.60 1/k0 window. In the experiments described here, the mass spectrometer was operated in PASEF DIA mode with exclusion of single-charged peptides. The DIA acquisition scheme consisted of 16 variable windows ranging from 400 to 1200 m/z.

#### Protein identification

Database searching and LFQ quantification (using XIC) was performed using DIA-NN (version 1.8.1; [31]). An updated UniProt *Rattus norvegicus* database was used for library-free search/library generation. For retention time prediction and extraction mass accuracy, we used the default parameter 0.0, which means that DIA-NN performed automatic mass and retention time correction. Top six fragments (ranked by their library intensities) were used for peptide identification and quantification. The false discovery rate (FDR) was set to 1% at the peptide precursor level. The variable modifications allowed were as follows: Nterm-acetylation and oxidation (M). In addition, C-propionoamide was set as fix modification. “Trypsin/P” was selected. Data were filtering according to a FDR of 1%. Cross-run normalization was performed using retention time-dependent.

#### Identification of differentially expressed proteins

To quantify the relative levels of protein abundance between different groups, data from DIA-NN were then analyzed using DEP package from R. Briefly, proteins that are identified in two out of three replicates of at least one condition were filtered, missing data were imputed using random draws from a manually defined left-shifted Gaussian distribution and differential enrichment analysis was based on a protein-wise linear models combined with empirical Bayes statistics. A log2FC 1.2 increase in relative abundance and a 0.01 *p* value were used to determine enriched proteins.

#### Enrichment analysis

Enrichments in biological process (BP) and pathways (KEGG) were performed using ClueGo App from Cytoscape software. Network specificity was set to medium; GO tree interval was set between 3 and 8. Cluster was performed using a selection set to 3-min genes and 4%. Enrichments were performed using a Bonferroni step-down method.

## Results

### In vitro studies on the expression of HIFs and their target genes in the H2030-BrM3 cells

To first evaluate if H2030-BrM3 cells are able to respond to a hypoxic microenvironment, we studied hypoxia-inducible factors (HIF-1 and HIF-2) and their target genes (*CA-IX, VEGFA, SLC2A1, CCDN1, SERPINE1 and TUBB3*) expressions under 1% of O_2_.

**Fig. 1:**
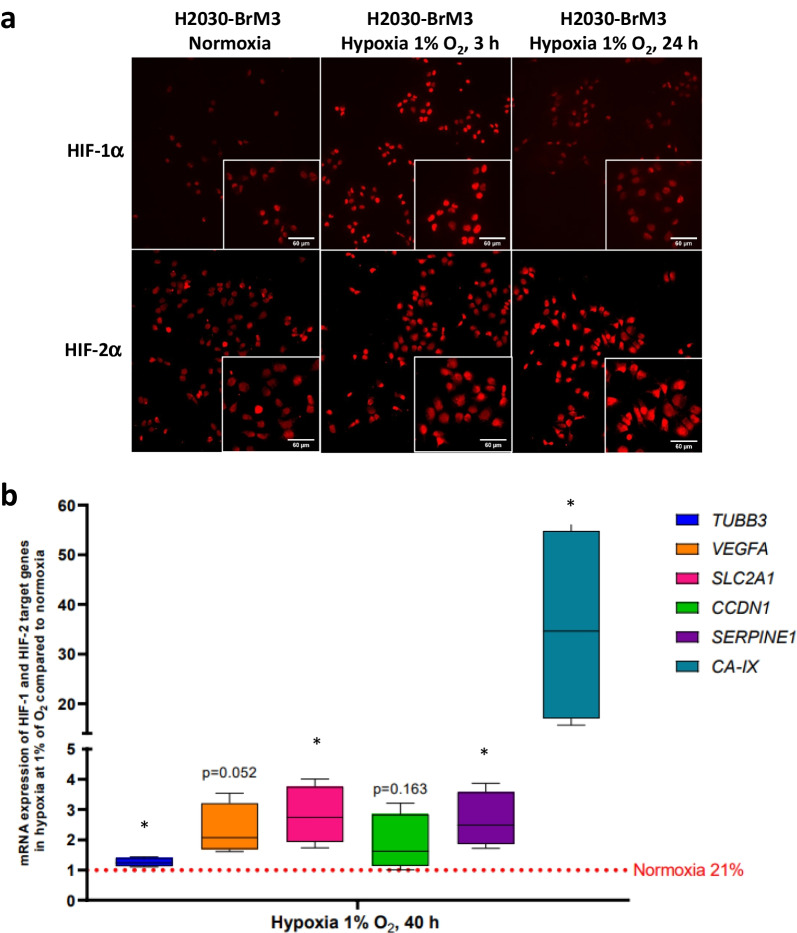
Effect of hypoxia on the expression of **a** HIF-1α, HIF-2α and **b** several HIF-target genes in H2030-BrM3 cells. **a** Immunocytology for HIF-1α and HIF-2α evaluated after 3 h or 24 h in hypoxic or normoxic condition. **b** Gene expression of HIF-target genes evaluated by real-time RT-PCR (TUBB3, VEGF-A, SLC2A1, CCDN1, SERPINE1 and CA-IX) in H2030-BrM3 cells submitted or not to hypoxia (1% O2) during 40 h. Median ± IQR (Interquartile Range), *n* = 4 independent experiments, * *p* < 0.05, one sample t-test vs theorical value of 1

Immunocytological labeling performed on H2030-BrM3 cells submitted to normoxia (21% O_2_) or hypoxia (1% O_2_) for 3 h and 24 h showed an increase in protein expression of both HIF-1α and HIF-2α isoforms under hypoxia. Indeed, an increase in HIF-1α and HIF-2α expression is observed after 3 h of hypoxia, which is still sustained for only HIF-2α after 24 h (Fig. 1a). Those observations could be linked to the “HIF switch,” a model proposed in which HIF-1α was described to drive the initial response to hypoxia whereas HIF-2α plays a major role in maintaining the hypoxic response during chronic exposure to hypoxia a phenomenon that we also observed in glioblastoma cell lines [28, 32].

We showed, by real-time RT-qPCR, in H2030-BrM3 cells submitted to hypoxia, increase mRNA expression of different well-known HIF-target genes including *TUBB3* (1.3-fold increase, *p* < 0.05), *SLC2A1* (2.8-fold increase, *p* < 0.05), *SERPINE1* (2.6-fold increase, *p* < 0.05) and *CA-IX* (35.5-fold increase, *p* < 0.05). A trend for *VEGF-A* gene expression is also observed (2.3-fold increase, *p* = 0.052) (Fig. 1b).

### Proteomic analyses in the H2030-BrM3 lung-derived brain metastasis model

To then study the features of TME of BM, we performed a proteomic approach in the BM model with the human lung adenocarcinoma-derived H2030-BrM3 cells implanted into the cortex and striatum of nude rats. Proteomic analyses were performed 24 days after tumor cell inoculation (D24). BM samples contain both human proteins from tumor-implanted cells (H2030-BrM3) and rat proteins from cells present in the TME (such as glial cells, inflammatory cells, etc.). Here, we aimed to compare protein expression changes in BM compared to healthy brain tissue, and therefore, only rat analyses are detailed in the results part. However, it is important to note that these analyses cannot distinguish accurately proteins expressed by TME and/or tumor cells themselves as numerous identical peptide sequences exist between rat and human. In total, 8420 proteins were quantified from the eight samples analyzed.

### Global protein expression analyses in the H2030-BrM3 lung-derived brain metastases

**Fig. 2:**
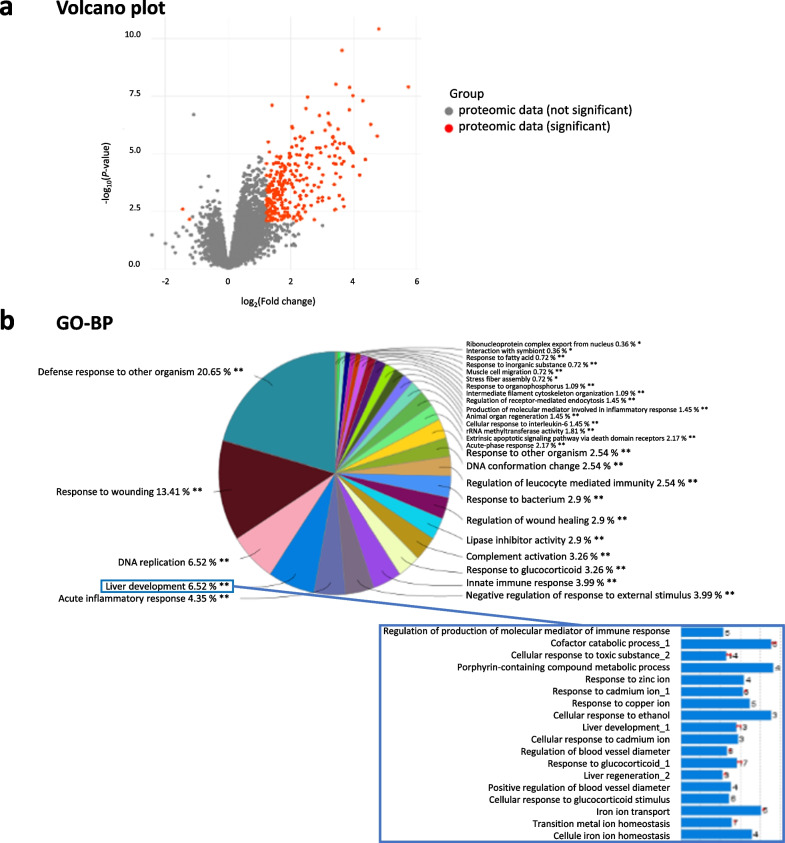
Global proteomic analyses in H2030-BrM3 lung-derived brain metastasis (BM) vs healthy brain tissues. **a** Volcano plots of quantified proteins between BM vs healthy brain tissues. Significant differentially expressed proteins are shown in red, cut-off: fold change (FC) = log2(1.2) (FC = 2.3) and *p* < 0.01, Bonferroni test. **b** GO enrichments of differentially expressed proteins on biological processes (BP). **FDR 1%, *FDR 5%. *n* = 5 for BM and *n* = 3 for healthy brain tissues

In a first step analysis, we combined the datasets of both cortical and striatal BM and those of healthy cortex and striatum tissues to explore the global tumor/stroma changes. This first analysis revealed, in particular, expression changes of proteins with known function in inflammation/immunity, DNA/RNA/protein processing/cell cycle, metabolism/oxidative stress/metal response, extracellular matrix/cytoskeleton/endothelium/wound healing and cell death processes (Fig. 2).

More precisely, a total of 275 accessions (protein groups) were found to be differentially expressed (*p* < 0.01) in BM compared to healthy tissues with 273 proteins up-regulated and two down-regulated more than log2FC of 1.2 (i.e., FC of 2.3) (Additional file 1: Table S1, Fig. 2a). Gene ontology (GO) enrichment analysis of all protein groups identified globally 29 groups of biological process (BP) (Fig. 2b). Of note, among GO groups, terms linked to response to metal ion and oxidative stress/oxygen species are retrieved (Fig. 2b, insert). For example, subunit of cytochrome c oxidase (CYP2J3), peroxidasin 1 (PXDN), heme oxygenase 1 (HMOX1 also named HO-1), hemoglobins (HBA-A3, HBB), subunit of ATP synthase (ATP5HL1), aldehyde dehydrogenases (ALDH3A1, ALDH2), adenylosuccinate synthetase isozyme 1 (AMPSase 1 also named Adssl1), UDP-glucuronosyltransferases (UGT1A1, UGT1A7), phospholipases (PLCG2, PLD4), carbamoyl-phosphate synthase (CPS1 also named CPSase 1) and glucosamine 6-phosphate N-acetyltransferase (GNPNAT1). Results also showed significant increase in ceruloplasmin (CP), albumin (ALB) and other metalloenzymes including MT-1, MT-2 and transferrin (TF) in BM compared to healthy brain tissues (Additional file 1: Table S1). This list is not exhaustive, see Additional file 1: Table S1 for all protein changes.

Besides increase in proteins involved in metabolic/oxidative pathways, the proteomic study revealed well-known protein expression changes in BM in cancer including lung cancers (CDK6, RAC2, integrin subunit alpha 3, FN1…), inflammation and immunity and in particular in the complement cascade (C1q A, C1q B, C1q C, C3, C4, C9, CfH and CfB), fibrinogens (FGA, FGB and FGG), plasminogen (PLG) and tissue-type plasminogen activator (tPA), serpins (SERPINA3K, SERPINA3N, SERPINA3C, SERPINA3L, SERPINA1; SERPING1; SERPINC1, SERPIND1), kininogen-1 (KNG1) and haptoglobin (HP) (Additional file 1: Table S1). Several proteins of the extracellular matrix and cytoskeleton are also increased such as GFAP, vimentin, fibronectin 1 (FN1), keratins (KRT8, KRT18 and KRT35), integrins (ITGA3, ITGB2 and ITGB4 also named GP150 or CD104, ITGAL) and filamin A (Additional file 1: Table S1).

Changes in protein expression involved in cell cycle, DNA replication and reparation (CDK1, 6, 7, PCNA, PARP14, POLD1, MCM2, -3,-4,-6,-7, MSH6, DNAJA2, TOP2A, PRKDC, RPLP1…), ribosome biogenesis (BOP1, EMG1, MRTO4, SPATA5, NSA2, RRS1…) and cell death including apoptosis (PDCD4, BAG1 and XAF1) are also observed (Additional file 1: Table S1).

In addition, several S100 proteins known to regulate multiple pathways including proliferation, differentiation, inflammation, migration and/or invasion, apoptosis, Ca^2+^ homeostasis and energy metabolism are increased in BM (S100A4,-A6,-A11) (Additional file 1: Table S1) [33].

Among proteins that are significantly increased in BM, it is important to highlight that several proteins are known to be involved in the HIF signaling pathways including HMOX1, PLCG2, TF, CP, KRT18, FN1 and HP (Additional file 1: Table S1) [34–37].

### Proteomic analysis of cortical BM versus striatal BM in the H2030-BrM3 lung-derived brain metastasis model

A second step of analysis was performed to potentially depict BM heterogeneities between cortical and striatal BM [13]. Therefore, this second comparison aimed to identify specific protein expression changes between cortical and striatal BM *vs* their respective healthy tissues. For full comparison of cortical BM versus healthy cortex and striatal BM versus healthy striatum, see Additional file 1: Table S2A and B, since only focus on main differences observed between cortical and striatal BM which are presented thereafter.

**Fig. 3:**
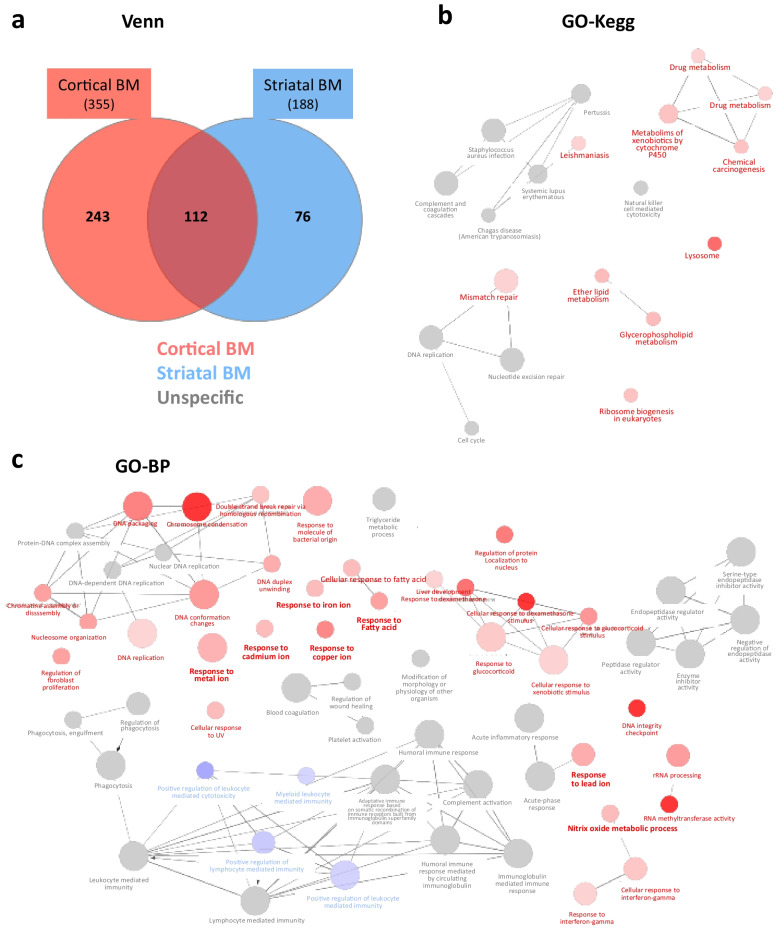
Proteomic analyses in cortical and striatal H2030-BrM3 lung-derived BM vs their respective healthy tissues. **a** Venn diagrams (Venn) of differentially expressed proteins identified from BM versus healthy tissues, from both cortical and striatal parts. **b, c** GO enrichment, using a Bonferroni’s method, in Kegg (**b**, GO-Kegg) and BP (**c**, GO-BP). Functions are shown in red for those preferentially enriched in cortical BM, blue in striatal BM and gray for unspecific (both in cortical and striatal BM). *n* = 5 for cortical and striatal BM, *n* = 3 for healthy brain tissues

The analysis showed 243 accessions specifically changed in cortical BM, 76 in striatal BM and 112 common in both BM (Fig. 3). Interestingly, GO enrichment analyses showed differences between cortical and striatal BM overall in DNA, RNA, cell cycle, cell signaling and inflammation but also in metabolism, oxidative, metal responses related-BP and pathways (Kegg) (Fig. 3 and Additional file 1: Table S3). In line with this more pronounced oxidative stress/hypoxia, metabolism and metal responses in cortical BM, an increase was observed for CP, TF, glutathione transferases and peroxidase (GSTM2 and MGST1), oxidoreductases including oxidase (COX4I2), dehydrogenase (ALDH3B1), haptoglobin (HP), acyl-CoA-related enzymes (HACL1) and nuclear respiratory factor 1 (NRF-1) (Additional file 1: Table S3) in cortical BM compared to striatal BM. Moreover, some of the S100 family proteins are exclusively overexpressed in cortical BM (S100A1,-A6) (Additional file 1: Table S3).

Overall, these proteomic studies suggest the presence of hypoxia and active redox metabolism in BM, which are more pronounced in cortical BM compared to striatal BM sustaining the potential interest of PET [^64^Cu][Cu(ATSM)] imaging not only to characterize TME of BM but also to depict inter-metastasis heterogeneity.

### In vivo PET/MRI imaging studies in the H2030-BrM3 lung-derived brain metastasis model

**Fig. 4:**
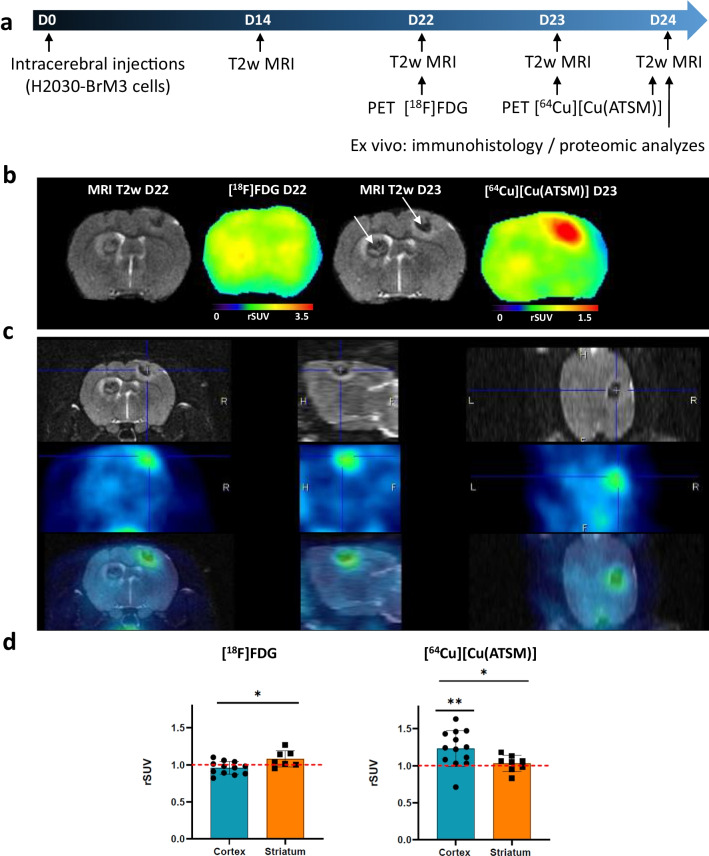
In vivo uptake of [^18^F]FDG and [^64^Cu][Cu(ATSM)] in the H2030-BrM3 BM model. **a** Timeline of experimental protocol. **b** Representative images of anatomical T2w MRI, [^18^F]FDG PET (1-h post-injection) and [^64^Cu][Cu(ATSM)] (4-h post-injection), white arrows represent the intratumoral hemorrhage. **c** MRI and [^64^Cu][Cu(ATSM)]PET, in the three planes (coronal, sagittal and transversal sections) acquired at D23. **d** Quantification of [^18^F]FDG, [^64^Cu][Cu(ATSM)] uptake into cortical BM and striatal BM (at 1-h and 4-h post-injection, respectively). Mean ± SD, *n* = 12 rats for cortical BM and *n* = 8 rats for striatal BM for [^18^F]FDG; *n* = 13 rats for cortical BM and *n* = 8 rats for striatal BM for [^64^Cu][Cu(ATSM)]. *p < 0.05 and **p < 0.01, one sample t-test vs theorical value of 1 and Mann–Whitney for comparison rSUV between cortical BM and striatal BM

Herein, we studied hypoxia/redox metabolism changes with [^64^Cu][Cu(ATSM)], and glucose metabolism with [^18^F]FDG in the BM model with the lung adenocarcinoma-derived H2030-BrM3 cells implanted into the cortex and striatum of nude rats. Anatomical MRI (1–2 weekly) was performed to monitor tumor development, followed by [^18^F]FDG PET and [^64^Cu][Cu(ATSM)] PET realized at 22 and 23 days after tumor cell inoculation (D22 and D23, respectively) (Fig. 4a).

Based on the anatomical T2w images, cortical BM had a mean tumor volume of 21.60 ± 18.4 mm^3^ at D22 and 40.1 mm^3^ ± 34.8 mm^3^ at D24 (Additional file 2: Figure S1a). For striatal BM, tumor development was slower: The mean tumor volume was 10.8 mm^3^ ± 9.4 mm^3^ at D22 and reached 12.3 mm^3^ ± 5.6 mm^3^ at D24 (Additional file 2: Figure S1a). Of note, a larger tumor volume in cortical BM compared to striatal BM has been observed at D24 (40.1 mm^3^ ± 34.8 mm^3^ for cortical BM versus 12.3 mm^3^ ± 5.6 mm^3^ for striatal BM, *p* < 0.05) (Additional file 2: Figure S1a). Tumor edema as well as intratumoral hemorrhage (white arrow) were observed in BM (Fig. 4b). Next, we studied glucose metabolism with [^18^F]FDG uptake at D22. No increase in glucose consumption within tumors has been observed, whatever the BM location (Fig. 4b). Indeed, the relative SUV (rSUV) is 0.96 ± 0.09 for cortical BM (with *p* = 0.1174, not significantly different from 1) and 1.08 ± 0.11 for striatal BM (with *p* = 0.1061 not significantly different from 1) (Fig. 4d, left part). Nevertheless, we could note a significant differential uptake between cortical and striatal BM (*p* < 0.05).

Then, BM were characterized in terms of hypoxia and redox metabolism changes through the use of [^64^Cu][Cu(ATSM)] radiotracer. The PET images obtained 4-h post-injection of [^64^Cu][Cu(ATSM)] at D23 revealed an uptake in cortical BM (Fig. 4b and c) within the tumor. Interestingly, we noticed the presence of an inter-metastasis heterogeneity with [^64^Cu][Cu(ATSM)] uptake between cortical and striatal BM since no significant uptake has been observed in striatal BM (Fig. 4b and c). Quantitatively, this preferential uptake of cortical BM is confirmed with an rSUV of 1.23 ± 0.24 (Fig. 4d, right part) significantly different from 1 (*p* < 0.01), while the rSUV of striatal BM is 1.03 ± 0.11 and not significant from 1 (*p* = 0.4629). Moreover, the inter-metastasis heterogeneity observed on PET imaging is confirmed by a significant difference in the uptake of cortical and striatal BM (*p* < 0.05). Of note, images taken 24 h after injection of [^64^Cu][Cu(ATSM)] (i.e. at D24) revealed not only uptake by cortical BM (rSUV of 1.21 ± 0.19), but also a significant uptake by striatal BM (rSUV of 1.19 ± 0.10) (Additional file 2: Figure S1b). Accordingly, the heterogeneity observed between cortical BM and striatal BM with [^64^Cu][Cu(ATSM)] at 4-h post-injection was not found any more significant 24 h after the radiotracer injection.

### Ex vivo studies of hypoxia and copper transporters in the H2030-BrM3 lung-derived brain metastasis model

**Fig. 5:**
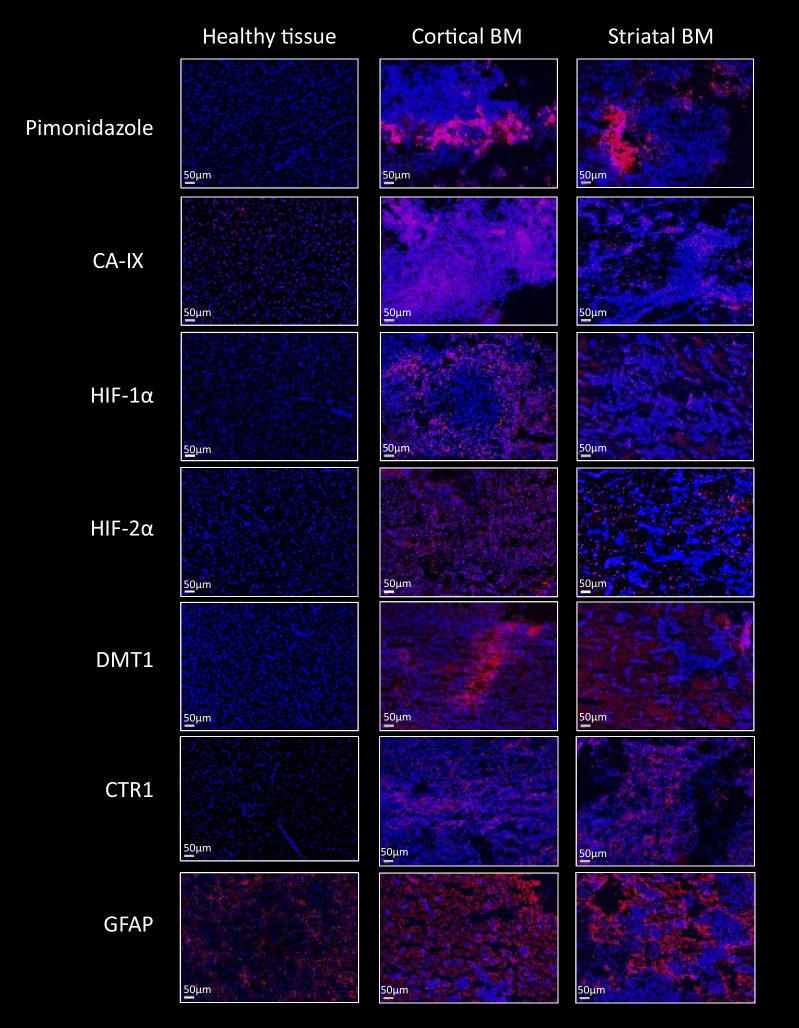
Immunohistological studies in cortical and striatal BM for hypoxia-related proteins, copper transporters and astrogliosis (red) with Hoechst 33,342 nuclear counterstaining (blue). Representative images of pimonidazole, CA-IX, HIF-1a, HIF-2a, DMT1, CTR1 and GFAP immunostaining in BM and healthy brain tissue

To confirm the presence of hypoxia suggested by the proteomic and PET results, we sought to further characterize the microenvironment of the BM by ex vivo approaches. After the last PET imaging, animals were euthanized, and their brains were harvested to perform immunohistofluorescence for pimonidazole, HIF-1α, HIF-2α and CA-IX as readout of hypoxia (Figs. 4a and 5). Pimonidazole, HIF-1α, HIF-2α as well as CA-IX immunostainings were positive in both cortical and striatal BM compared to healthy brain tissue confirming the presence of hypoxia in the TME of BM (Fig. 5). Then, as some metal transporter expressions are also known to be increased by hypoxia (via HIF) [26, 38], we also studied expression of the specific copper transporter 1 (CTR1), the main copper cell transporter as well as the non-specific divalent metal transporter 1 (DMT1) [39, 40]. We observed a positive labeling of those transporters in both cortical and striatal BM (Fig. 5). Lastly, as astrocytes have been described as a cornerstone in copper metabolism in the brain [41], we performed glial fibrillary acidic protein (GFAP) immunostaining revealing an astrocytic activation in both cortical and striatal BM (Fig. 5).

Overall, [^64^Cu][Cu(ATSM)] PET imaging, immunohistological and proteomic results showed the presence of hypoxia and active redox metabolism in BM, which are more pronounced in cortical BM compared to striatal BM.

## Discussion

Human non‑small cell lung cancers (NSCLC) are associated with an extremely poor prognosis especially for the 50% of patients developing BM despite several therapeutic strategies including whole-brain or stereotactic radiotherapy combined or not with new systemic targeted therapies [42]. Tumor hypoxia is commonly associated with malignant progression, metastasis, resistance to chemo- and/or radiotherapy, recurrence and overall poor prognosis including in lung cancers [8, 13, 43]. Therefore, detection of tumor hypoxia is of great importance to optimize the treatment strategy and improve overall prognosis.

PET imaging of hypoxia with [^18^F]FMISO has been widely used in the past [44–46]. While [^64^Cu][Cu(ATSM)] was originally developed to visualize hypoxic regions, it is now rather admitted that its accumulation in tumor cells is also related to the over-reduced cellular state and thus proposed as a promising imaging radiotracer for the detection of oxidative stress [18–20]. Indeed, several studies have shown that it can accumulate in normoxic tissues where oxidative stress can be induced by a variety of causes including mitochondrial dysfunction, inflammation and hypoxia itself [18]. Of note, reactive oxygen species (ROS) and nitric oxide (NO) can, in turn, inactivate prolyl hydroxylase-2 (PHD2), which participate to further increase HIFs activation, the major oxygen sensors [47].

Herein, we showed for the first time, using a H2030-BrM3 lung-derived BM model in rats, that [^64^Cu][Cu(ATSM)] could be interesting to complete the arsenal of BM imaging. In particular as we confirmed with both immunohistochemical and proteomic approaches that TME of BM is hypoxic and presents metabolic/oxidative changes that can be linked not only to hypoxia but also to inflammation known to occur in BM [48]. Indeed, pimonidazole, HIFs and CA-IX immunostaining confirmed that BM developed in this preclinical model are hypoxic along with a glial reaction. In vitro analyses also showed that H2030-BrM3 cells further express HIFs and their target genes (such as *TUBB3, VEGF-A, SLC2A1, CA-IX, CCDN1 and SERPINE1*) under hypoxic conditions. Interestingly, the proteomic study showed that numerous proteins involved in metabolism, oxidative stress, oxidative phosphorylation and metal response (HMOX1, ALDH3A1, ALDH2, CP, ALB, MT-1, MT-2, TF…) are increased in BM. Aldehyde dehydrogenases (ALDHs), a group of enzymes that catalyze the oxidation of aldehydes to less toxic carboxylic acids and which have been reported to mediate the acquired drug resistance of tumor cells, as well as hyperactive glutathione (GSH) metabolism pathway were also previously found to be up-regulated in BM from NSCLC [49]. This result is also in accordance with those of You et al. [50] showing that high expression of ALDH1A2 mRNA was found to be significantly correlated to worsen overall survival in all NSCLC patients. Moreover, proteins known to be involved in the HIF-1 signaling pathways such as HMOX1, PLCG2, TF, CP, KRT18 and FN1 [34, 36, 51] are also increased in BM. These results are in accordance with those of Wei et al*.* [52] showing that gene sets associated with oxygen-related metabolism, such as hypoxia, glycolysis, oxidative phosphorylation and reactive oxygen species pathways are significantly enriched by brain metastatic lung tumor cells and might confer their phenotypic plasticity.

Of interest, an increase in ceruloplasmin (CP) has been also showed from the proteomic study in BM (3.70-fold increase). CP, the primary copper transporter in the blood, is a ferroxidase [53, 54]. In the central nervous system, CP is predominantly expressed by astrocytes [53]. It plays an essential role in iron homeostasis thought the conversion of ferrous iron Fe^2+^ to ferric iron Fe^3+^ which is internalized by cells via TF and therefore regulates ferroptosis in cancer cells. As the early 1984s, it has been shown in patients with primary brain tumors an increase in serum copper and CP levels that potentially associated with decreased catabolism of CP [55, 56]. Due to its ferroxidase activity, CP has a role in the management of oxidative stress. High levels of CP led to increased production of ROS leading to DNA damage induced by hydrogen peroxide or releasing copper ions [57–59]. Moreover, recently, Roy et al*.* [57] showed on two human glioblastoma cell lines (U251 and U87), a role for CP in the control of cell responses to radiation. Of note, besides that of CP, expression of other metalloenzymes including TF, MT-1 and MT-2 is also increased in BM in the present study, all described to up-regulated by HIFs and associated to carcinogenesis and cancer treatment resistance [60].

The proteomic study also revealed numerous protein expression changes in inflammation known to occur in BM and in particular in the complement cascade in addition to endothelium/extracellular matrix/cytoskeleton/wound healing-related proteins [48]. Extracellular matrix molecules can activate paracrine or autocrine cell signaling remodel tissue architecture during inflammation creating a favorable environment for cancer development. Those modifications are in accordance with BM inducing activation of microglia/macrophage resident cells as well as the recruitment of immune and inflammatory cells from the periphery [61]. However, it is noteworthy that in our study, the animals used are nude rats due to the use of human H2030-BrM3 cells. This strain of rats is characterized by a deficient immune system due to an insufficient production of T cells. Thus, the immune response may be reduced with respect to T lymphocytes in this preclinical model.

All of these protein expression changes and in particular those related to metabolic/oxidative/metal responses that can be linked not only to hypoxia but also to inflammation underline that [^64^Cu][Cu(ATSM)] imaging could be of interest for the therapeutic management of BM. Indeed, we showed for the first time on a H2030-BrM3 lung-derived BM model in rats that [^64^Cu][Cu(ATSM)] uptake is increased in BM. Interestingly, besides passive penetrance of the [^64^Cu][Cu(ATSM)] in cells, expression of Cu-dependant transporters, that some can be further increased by hypoxia (as CTR1, DMT1), could contribute to [^64^Cu][Cu(ATSM)] uptake in tumors, as already shown in the previous studies on glioblastoma [26, 62]. Accordingly, disturbances in Cu transporters change Cu trafficking and Cu-containing enzymes, all of which are involved in tumor progression and metastasis including in lung cancers [63–65]. It is known that tumor cells itself but also other cells of TME such as endothelial, glial and inflammatory cells express Cu transporter and Cu-containing enzymes which may participate to copper accumulation into the BM region [66, 67]. Although the global proteomic approach did not reveal significant expression changes of DMT1 and CTR1 in BM compared to brain healthy tissues, our immunohistochemistry study confirmed their presence in BM. Moreover, the proteomic study, as mentioned before, revealed important increase in CP as well as TF and other metalloproteins such as MT-1 and MT-2 in BM.

Besides CP, the proteomic analysis between cortical and striatal BM revealed other proteins involved in metabolism/oxidative phosphorylation/oxidative stress/metal response. For example, glutathione S-transferase Mu 2, GSTM2, an enzyme involves in metabolism and/or detoxification of various endogenous metabolites appeared more abundant in cortical BM compared to striatal BM. This is line with several studies underlying GSTM2 has a chemoresistance marker including in lung cancer [43, 68]. Haptoglobin (HP), a blood plasma glycoprotein, plays a critical role in tissue protection, and the prevention of oxidative damage is also overexpressed in cortical BM compared to striatal BM. Overexpression of HP has been found in lung cancer, ovarian and breast cancers, as well as in glioblastoma and metastases [69, 70]. Moreover, these results are in accordance with those of Wang et al*.* [71] showing, in glioblastoma, that high expression of both ALDH3B1 could influence tumor cell proliferation and migration. In addition, many S100 proteins, known to be implicated in cancer development and metastasis, are increased in BM, and some of them, like S100 A6, are further increase in cortical BM vs striatal BM [72–74]. These results are also in line with a previous study showing that transcription of S100 A6 gene is increased by agents known to evoke oxidative stress [75]. Aquaporin 1 (AQP1) is also more abundant in cortical BM which is in line with several studies suggesting that aquaporins contribute to motility, invasiveness and edema formation and facilitate metabolism in tumor cells under hypoxic conditions [76, 77].

While we showed that [^64^Cu][Cu(ATSM)] could be interesting to complete the arsenal of BM imaging, only cortical BM are significantly positive at D23. These results are in line with the [^18^F]FMISO PET results underlying that cortical BM might be more hypoxic compared to striatal BM [13]. This effect could be due to vascularization differences as previously observed by immunohistochemistry [13] and/or over oxidation–reduction, in cortical BM compared to striatal ones, which can be also induced by hypoxia. Moreover, it is known in the literature that BM develop preferentially in the cortex compared to deep brain structures [78, 79]. These results are also in agreement with preclinical study showing higher expression of acetyl-CoA content a central metabolite, in healthy cortex compared to healthy striatum, a difference that is maintained in the presence of BM which could explain a developmental difference between these two structures [13, 80]. In our present study, we also find acyl-CoA-related proteins enriched in cortical BM compared to striatal BM. These differences in metabolism/nutrients between the brain regions may differentially influence cancer cells, for example in terms of proliferative rate, as underlined by the literature, and our previous results obtained by [^18^F]FLT (3′-deoxy-3′-[18F]-fluorothymidine) PET analyses [13, 81]. Therefore, another explanation of the difference in uptake of [^64^Cu][Cu(ATSM)] in BM at D23 could be due to a larger tumor volume in cortical BM compared to striatal BM. However, if a significant difference in tumor volume between cortical BM and striatal BM is observed at D24, none is observed at D23. Moreover, additional data obtained at D24, i.e., 24 h after [^64^Cu][Cu(ATSM)] injection, showed a significant tracer uptake by both cortical BM and striatal BM without significant difference between the BM. In addition, an absence of correlation between [^64^Cu][Cu(ATSM)] uptake and tumor volume at D23 has been observed (data not shown). Altogether, these data suggest that the difference of [^64^Cu][Cu(ATSM)] uptake between cortical and striatal BM at D23 may not be due to difference between tumor volumes.

Of note, the present work also underlines that late [^64^Cu][Cu(ATSM)] PET imaging could be more influenced by the copper metabolism than short PET imaging. Indeed, as discussed in a review of Liu et al. [20], transport of copper from blood uptake contains two phases: a first phase after injection where copper will be absorbed rapidly by ALB and transcuprein which deliver copper to CTR1, reaching a minimum level in plasma within approximately 2 h and a reemergence phase of copper in plasma which starts from 6 h to approximately 1 day after initial injection at which point the blood copper concentration reaches another maximum, this time incorporated with CP and being transported to other tissues. Therefore, the increase in CP expression observed in both cortical BM and striatal BM (with a more pronounced one in cortical BM) could explain that, after a delayed acquisition (24 h after [^64^Cu][Cu(ATSM)] injection, i.e., at D24), striatal BM became also positive.

Overall, the present work, on the H2030-BrM3 lung-derived BM model in rats, confirmed the presence of hypoxia and protein expression changes linked to hypoxia and oxidative stress in the BM microenvironment [13]. More importantly, it showed for the first time the interest of [^64^Cu][Cu(ATSM)] PET together with other multimodal PET/MRI imaging to detect tumor growth, hypoxia-oxidative changes that could be of use to depict inter-metastasis heterogeneity as well as to guide treatments. Indeed, in clinical studies, Cu-ATSM has been shown to be predictive of response to traditional cancer therapies in patients with rectal, lung and uterine cervix cancer, while in these same studies, concurrent imaging with [^18^F]FDG showed no predictive value [82–84]. These results together with our results with [^18^F]FDG PET are in accordance with the complementarity of multimodal imaging. A study comparing [^18^F]FMISO and [^18^F]FDG uptake in humans highlights that some tumors can be hypoxic and have moderate glucose metabolism, and conversely, some tumors with high metabolism are not hypoxic [23].

Radiotracers such as [^64^Cu][Cu(ATSM)] that particularly allows detecting oxidative changes could be of interest to detect cancer treatment resistance and guide treatments. For example, a potential interest of Cu-ATSM has been recently shown for carbon ion therapy since relative biological effectiveness (RBE) of carbon ions has been shown to be associated with [[^64^Cu][Cu(ATSM)] uptake and with antioxidant capacity in cancer cells. These new findings highlight the potential utility of Cu-ATSM imaging to identify high RBE tumors that will benefit from carbon ion therapy [85]. In addition, the copper transporter CTR1, as well as ATP7A and ATP7B, has been demonstrated to regulate the flow of cisplatin and its analog into the cell. Therefore, [^64^Cu][Cu(ATSM)] imaging which has been shown to have a good ability to detect NSCLC lesions may be useful to differentiate between those patients who may benefit from platinum-based therapy [86].

As well reported in the review of Xie and Wei [87], [^64^Cu][Cu(ATSM)], compared to other hypoxia-selective tracers, presents the advantage not only to reflect hypoxic changes but also over-reduced intracellular states caused by mitochondrial dysfunction that can be independent of hypoxia. Hypoxia and cellular redox status are two important interconnected phenomena modulating the cancer treatment response including that of chemo- and radiotherapy [88]. Therefore, a tracer that images both biological components could be of interest to guide but also predict and evaluate cancer treatment response in patients. Several clinical studies are done or are still ongoing in glioblastoma, lung, rectum and cervical cancers to evaluate the interest of [^64^Cu]Cu-ATSM to predict the treatment response [24, 87].

Importantly, another advantage of [^64^Cu][Cu(ATSM)] compared to other hypoxia tracers such as [^18^F]FMISO, in the context of brain imaging, relies on its enhanced permeability toward the blood–brain barrier (BBB). Comparatively to fluorinated PET agents, Cu-ATSM shows better contrast in hypoxia regions without metabolite accumulation in healthy tissues. Furthermore, hypoxia and redox status changes are also present in non-tumoral diseases like stroke or neurodegenerative disorders for which [64Cu][Cu(ATSM)] imaging could present potential interest especially as BBB is a major obstacle for radiotracers [87].

In addition, ^64^Cu-labeled agents are longer-lived radiopharmaceuticals that should facilitate shipping to multiple centers for multi-center clinical trials and therefore its use in clinics.

Finally, [^64^Cu][Cu(ATSM)] may be used not only as a PET imaging agent but also as an internal radiotherapy agent against tumors because ^64^Cu shows β + decay as well as β-decay and electron capture [89]. Indeed, there are also clinical studies evaluating the safety profiles and preliminary efficacies of Cu(II)ATSM in patients with Parkinson disease or amyotrophic lateral sclerosis/motor neuron disease [87].

## Conclusion

Collectively, from proteomic and imaging approaches done in the H2030-BrM3 lung-derived brain metastasis model, we showed major protein expression changes involved in metabolism and oxidative-related pathways known to be induced not only by hypoxia but also inflammation in TME and/or anti-cancer treatments. We then emphasized that [^64^Cu][Cu(ATSM)], which has never been studied in the BM context, may be useful to image oxidative changes in BM and to depict inter-metastasis heterogeneity that could be of clinical utility to refine treatment strategy. However, the real place of [^64^Cu][Cu(ATSM)] still requires to be defined but it appears to be important to cross different tumor information such metabolic activity (e.g., FDG), phenotypic information (e.g., antigen overexpression) and microenvironment information (such as hypoxia/redox status). In this extended radiomic picture, [^64^Cu][Cu(ATSM)] could play an important place in close future. Nevertheless, further translation studies are needed to fill the gaps including in the understanding of the mechanisms accounting for [^64^Cu][Cu(ATSM)] uptake and better address its clinical value for diagnostic and/or to guide the therapeutic scheme in particular in the context of BM. The clinical place of tumor hypoxia/redox status mapping in the patient care management must be considered in link with the recent radiotherapy progress such as radiotherapy enhancement with better dose distribution at cellular level, hadrontherapy (such as carbon therapy).

### Supplementary Information

**Additional file 1**. **Table S1**: quantitative label-free (XIC) rat proteomic analysis from BM (n = 5, cortical and striatal BM combined dataset) vs healthy brain tissues (n = 3, cortex and striatum combined dataset). LogFC and statistics were calculated from DEP R package (see Materials and Methods). **Table S2**: quantitative label-free (XIC) rat proteomic analysis from (A) cortical and (B) striatal BM (n = 5) vs their respective healthy tissues (n = 3). LogFC and statistics were calculated from DEP R package (see Materials and Methods). **Table S3**: common and differently expressed proteins from cortical and striatal BM vs healthy cortex and striatum (Venn diagram) from rat proteomic analysis. LogFC and statistics were calculated from DEP R package (see Materials and Methods).

**Additional file 2**.**Figure S1**: Comparison of tumor volume (in mm3) in both brain structures (cortex and striatum), at D22, D23 and D24 after intracerebral tumor cell implantation (a). Mean ± SD, n = 12 rats for cortical BM at D22, n = 13 rats for cortical BM at D23, n = 10 rats for cortical BM at D24 and n= 8 rats for striatal BM whatever the time studies. *p < 0.05, two-way ANOVA followed by Tukey’s test. (b) Quantification of [64Cu][Cu(ATSM)] uptake, 24-h post-injection, into cortical BM and striatal BM. Mean ± SD, n=10 for cortical BM and n=8 for striatal BM. One sample t-test vs theorical value of 1 and Mann-Whitney for comparison rSUV between cortical BM and striatal BM.

## Data Availability

The datasets analyzed during the current study, are stored at the CYCERON center and University of Caen and are available from the corresponding author on reasonable request. Proteomic data are available at iPROX IPX0005866001.

## Abbreviations

[^18^F]FDG: 2-[18F]fluoro-2-deoxy-d-glucose
[^18^F]FMISO: 1H-1-(3-[18F]fluoro-2-hydroxypropyl)-2-nitroimidazole
[^18^F]FLT: 3′-Deoxy-3′-[18F]fluorothymidine
[^64^Cu][Cu(ATSM)]: [64Cu][Cu-diacetyl-bis(N4-methylthiosemicarbazone)]
BM: Brain metastasis
CA-IX: Carbonic anhydrase IX
CTR1: Copper transporter 1
DMT1: Divalent metal transporter 1
HIF: Hypoxia-inducible factor
MRI: Magnetic resonance imaging
PET: Positron emission tomography
SUV: Standardized uptake value
rSUV: Relative SUV
TME: Tumor microenvironment
