# Supplementary figures and images for: Assessment of hypoxia and oxidative-related changes in a lung-derived brain metastasis model by [64Cu][Cu(ATSM)] PET and proteomic studies
Source: EJNMMI Res. 2023 Nov 25;13:102. doi: 10.1186/s13550-023-01052-8 (PMC10676347; doi:10.1186/s13550-023-01052-8)

## Slide 1
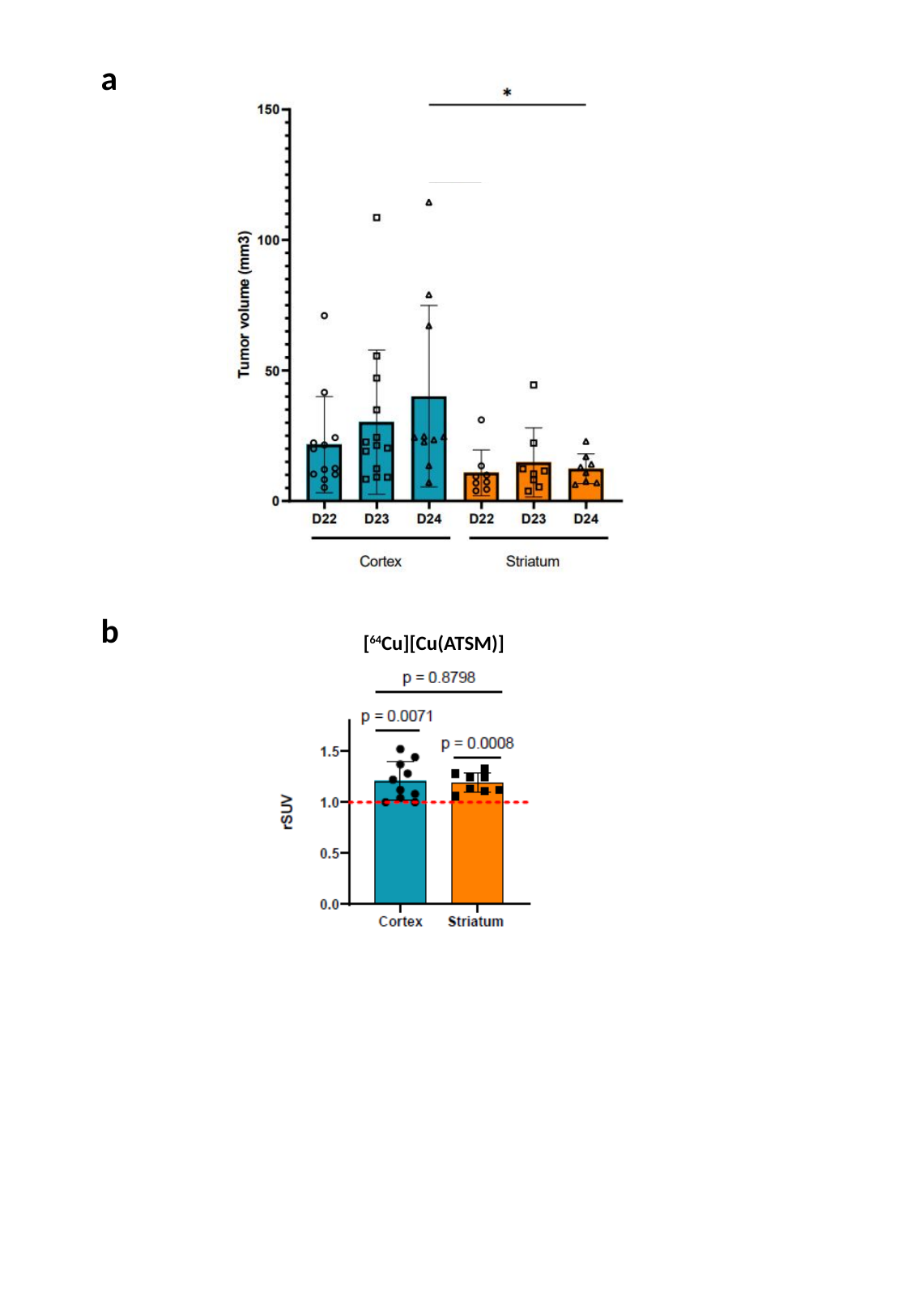

a
b
[64Cu][Cu(ATSM)]

Supplement: Supplementary file 2 — Additional file 2.Figure S1: Comparison of tumor volume (in mm3) in both brain structures (cortex and striatum), at D22, D23 and D24 after intracerebral tumor cell implantation (a). Mean ± SD, n = 12 rats for cortical BM at D22, n = 13 rats for cortical BM at D23, n = 10 rats for cortical BM at D24 and n= 8 rats for striatal BM whatever the time studies. *p < 0.05, two-way ANOVA followed by Tukey’s test. (b) Quantification of [64Cu][Cu(ATSM)] uptake, 24-h post-injection, into cortical BM and striatal BM. Mean ± SD, n=10 for cortical BM and n=8 for striatal BM. One sample t-test vs theorical value of 1 and Mann-Whitney for comparison rSUV between cortical BM and striatal BM. [file 13550_2023_1052_MOESM2_ESM.pptm]
